# 

**Published:** 1907-11-02

**Authors:** 


CONTENTS.

PAGE
**Disease and National Efficiency** - - - - - 103
**The Neglected Art of Prescription Writing** - - 104
**Annotations—**
The Spread of Disease by Living Agents—The Proof of Live-Birth—The Ophthalmological Society - - - - - 105
**Medical Opinion and Movement** - - - - - 106
**Hospital Clinics—**
Arterio-Sclerosis. By John Lindsay Steven, M.D. - - - 107
Fretfulness in Infancy. By William P. S. Branson, M.D., M.R.C.P. - - - - - - - - - - - - - - 110
**Special Article—**
The Third Report of the Royal Commission on Vivisection - 111
**Illustrative Cases—**
Post-Hemiplegic Athetosis - - - - - - - - - 113
Acute Yellow Atrophy of the Liver - - - - - - 113
Indigo-Uria - - - - - - - - - - - - 114
**Points in Surgery—**
The Treatment of Fractures from a Common-sense Point of View - - - - - - - - - - - - - - 115
Colostomy - - - - - - - - - - - - - 116

PAGE
**Gynæcology—**
The Common Form of Dysmenorrhœa of Young Women - - 117
**Ophthalmology—**
Injuries to the Eye - - - - - - - - - - - 118
**Points in Pathology—**
Pathology for General Practitioners - - - - - - - 119
The Prussian Blue Reaction of the Liver in Pernicious Anæmia 120
**The Practitioner's Relaxations and Hobbies—**
Archæology and the Collection of Antiques - - - - 121
**Practical Notes on Diagnosis and Treatment** - 122
**Therapeutics—**
Prescriptions and Dispensing - - - - - - - 123
**Hospital Administration—**
St. Mary's Hospital - - - - - - - - - - - 125
News and Coming Events - - - - - - - - - 124
The New Buildings at St. Bartholomew's Hospital - - - 126
**Editor's Letter-Box—**
The Temporary Relief of Turbinal Distension - - - - 126
**Nursing Administration—**
Committees and Justice - - - - - - - - - 127
The Common Task - - - - - - - - - - - 128

